# 5-Chloro-2,7-dimethyl-3-(3-methyl­phenyl­sulfon­yl)-1-benzo­furan

**DOI:** 10.1107/S1600536814007892

**Published:** 2014-04-16

**Authors:** Hong Dae Choi, Pil Ja Seo, Uk Lee

**Affiliations:** a Department of Chemistry, Dongeui University, San 24 Kaya-dong, Busanjin-gu, Busan 614-714, Republic of Korea; b Department of Chemistry, Pukyong National University, 599-1 Daeyeon 3-dong, Nam-gu, Busan 608-737, Republic of Korea

## Abstract

In the title compound, C_17_H_15_ClO_3_S, the dihedral angle between the mean planes of the benzo­furan and 3-methyl­phenyl rings is 76.99 (4)°. In the crystal, mol­ecules are linked by C—H⋯O hydrogen bonds into chains along the *b*-axis direction. These chains are linked by π–π inter­actions between the benzene and furan rings of neighbouring mol­ecules [centroid–centroid distance = 3.976 (2) Å].

## Related literature   

For background information and the crystal structures of related compounds, see: Choi *et al.* (2011 ▶, 2013 ▶). For the pharmacological activity of benzo­furan compounds, see: Aslam *et al.* (2006 ▶); Galal *et al.* (2009 ▶); Khan *et al.* (2005 ▶).
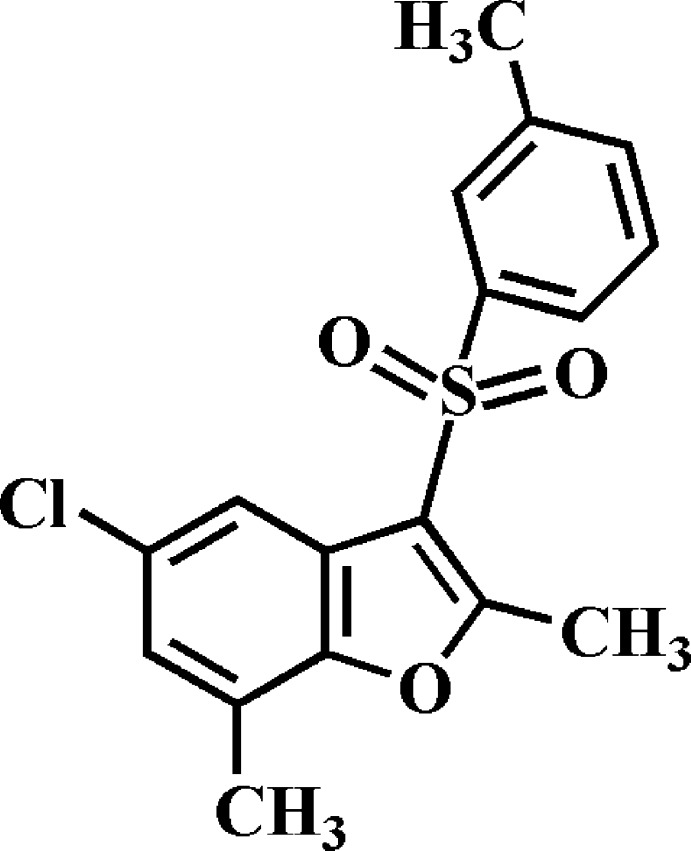


## Experimental   

### 

#### Crystal data  

C_17_H_15_ClO_3_S
*M* *_r_* = 334.80
Monoclinic, 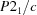

*a* = 8.8707 (2) Å
*b* = 6.5281 (2) Å
*c* = 26.3574 (6) Å
β = 96.998 (1)°
*V* = 1514.96 (7) Å^3^
*Z* = 4
Mo *K*α radiation
μ = 0.40 mm^−1^
*T* = 173 K
0.39 × 0.37 × 0.18 mm

#### Data collection  

Bruker SMART APEXII CCD diffractometer
Absorption correction: multi-scan (*SADABS*; Bruker, 2009 ▶) *T*_min_ = 0.379, *T*_max_ = 0.746
25926 measured reflections
3785 independent reflections
3252 reflections with *I* > 2σ(*I*)
*R*_int_ = 0.033

#### Refinement  

*R*[*F* ^2^ > 2σ(*F* ^2^)] = 0.035
*wR*(*F* ^2^) = 0.097
*S* = 1.04
3785 reflections
202 parameters
H-atom parameters constrained
Δρ_max_ = 0.32 e Å^−3^
Δρ_min_ = −0.36 e Å^−3^

### 

Data collection: *APEX2* (Bruker, 2009 ▶); cell refinement: *SAINT* (Bruker, 2009 ▶); data reduction: *SAINT*; program(s) used to solve structure: *SHELXS97* (Sheldrick, 2008 ▶); program(s) used to refine structure: *SHELXL97* (Sheldrick, 2008 ▶); molecular graphics: *ORTEP-3 for Windows* (Farrugia, 2012 ▶) and *DIAMOND* (Brandenburg, 1998 ▶); software used to prepare material for publication: *SHELXL97*.

## Supplementary Material

Crystal structure: contains datablock(s) I. DOI: 10.1107/S1600536814007892/im2452sup1.cif

Structure factors: contains datablock(s) I. DOI: 10.1107/S1600536814007892/im2452Isup2.hkl

Supporting information file. DOI: 10.1107/S1600536814007892/im2452Isup3.cml

CCDC reference: 996302

Additional supporting information:  crystallographic information; 3D view; checkCIF report

## Figures and Tables

**Table 1:** Hydrogen-bond geometry (Å, °) *Cg*1 and *Cg*2 are the centroids of the C2–C7 benzene ring and the C1/C2/C7/O1/C8 furan ring, respectively.

*D*—H⋯*A*	*D*—H	H⋯*A*	*D*⋯*A*	*D*—H⋯*A*
C10—H10*C*⋯O2^i^	0.98	2.49	3.297 (2)	139
C17—H17*A*⋯O3^ii^	0.98	2.41	3.369 (2)	165

Symmetry codes: (i) 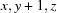; (ii) 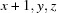.

**Fig. 1.**
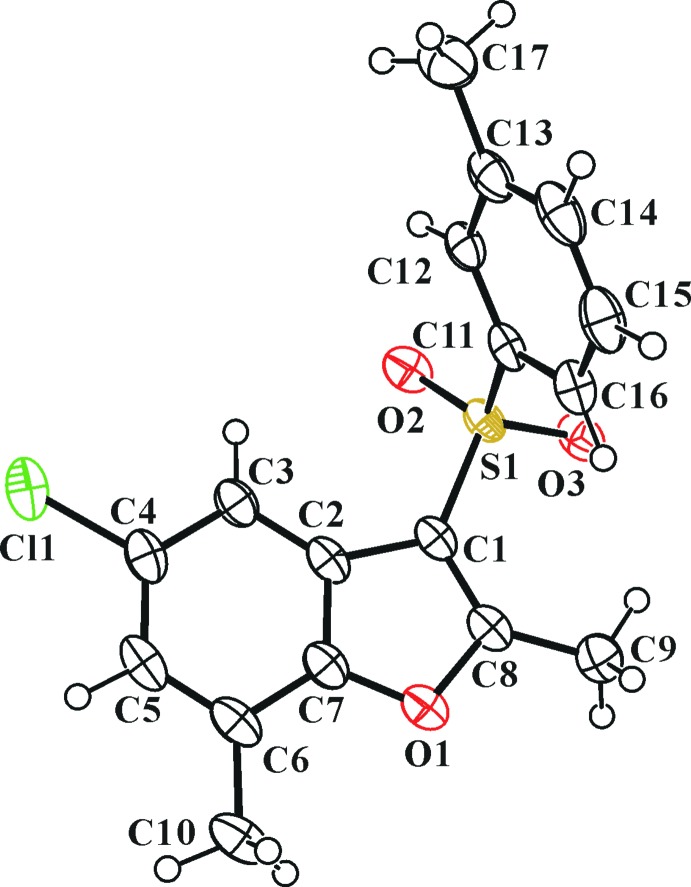
The molecular structure of the title compound with the atom numbering scheme Displacement ellipsoids are drawn at the 50% probability level. Hydrogen atoms are presented as small spheres of arbitrary radius.

**Fig. 2.**
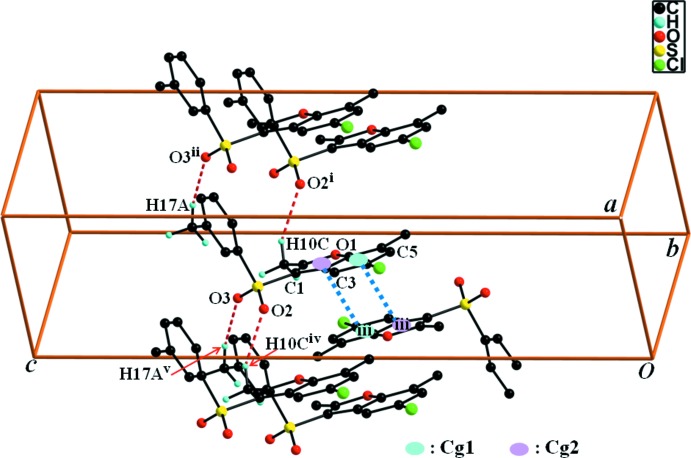
A view of the C—H..O and π···π interactions (dotted lines) in the crystal structure of the title compound. H atoms non-participating in hydrogen-bonding were omitted for clarity. [Symmetry codes: (i) x, y + 1, z; (ii) x + 1, y, z; (iii) - x, - y + 1, - z + 1; (iv) x, y - 1, z; (v) x - 1, y, z.]

## supplementary crystallographic information

### 1. Comment

Many compounds involving a benzofuran moiety have attracted much attention due to their valuable pharmacological activities such as antifungal (Aslam *et al.*, 2006), antitumor and antiviral (Galal *et al.*, 2009) and antimicrobial (Khan *et al.*, 2005) properties.

As a part of our ongoing study of 5-chloro-2,7-dimethyl-1-benzofuran derivatives containing cyclohexylsulfinyl (Choi *et al.*, 2011) and 4-bromophenylsulfinyl (Choi *et al.*, 2013) substituents in the 3-position, we report here on the crystal structure of the title compound.

In the title molecule (Fig. 1), the benzofuran ring system is essentially planar, with a mean deviation of 0.007 (1) Å from the least-squares plane defined by the nine constituent atoms. The 3-methylphenyl ring also is essentially planar, with a mean deviation of 0.004 (1) Å from the least-squares plane defined by the six constituent atoms. The dihedral angle formed by the benzofuran ring system and the 3-methylphenyl ring is 76.99 (4)°. In the crystal structure (Fig. 2), molecules are linked by C—H···O hydrogen bonds (Table 1) into chains along the *b*-axis direction. These chains are further connected by π···π interactions between the benzene and furan rings of neighbouring molecules, with a Cg1···Cg2^iii^ distance of 3.976 (2) Å and an interplanar distance of 3.470 (2) Å resulting in a slippage of 1.941 (2) Å (Cg1 and Cg2 are the centroids of the C2–C7 benzene ring and the C1/C2/C7/O1/C8 furan ring, respectively).

### 2. Experimental

3-Chloroperoxybenzoic acid (77%, 448 mg, 2.0 mmol) was added in small portions to a stirred solution of 5-chloro-2,7-dimethyl-3-(3-methylphenylsulfanyl)-1-benzofuran (272 mg, 0.9 mmol) in dichloromethane (40 mL) at 273 K. After being stirred at room temperature for 8h, the mixture was washed with saturated sodium bicarbonate solution and the organic layer was separated, dried over magnesium sulfate, filtered and concentrated at reduced pressure. The residue was purified by column chromatography (benzene) to afford the title compound as a colorless solid [yield 71%, m.p. 426–427 K; *R*_f_ = 0.52 (benzene)]. Single crystals suitable for X-ray diffraction were prepared by slow evaporation of a solution of the title compound in ethyl acetate at room temperature.

### 3. Refinement

All H atoms were positioned geometrically and refined using a riding model, with C—H = 0.95 Å for aryl and 0.98 Å for methyl H atoms, respectively. *U*_iso_ (H) = 1.2*U*_eq_ (C) for aryl and 1.5*U*_eq_ (C) for methyl H atoms. The positions of methyl hydrogens were optimized using the SHELXL-97's command AFIX 137 (Sheldrick, 2008).

### Crystal data

**Table:**

C_17_H_15_ClO_3_S	*F*(000) = 696
*M**_r_* = 334.80	*D*_x_ = 1.468 Mg m^−^^3^
Monoclinic, *P*2_1_/*c*	Melting point = 427–426 K
Hall symbol: -P 2ybc	Mo *K*α radiation, λ = 0.71073 Å
*a* = 8.8707 (2) Å	Cell parameters from 9774 reflections
*b* = 6.5281 (2) Å	θ = 2.3–28.2°
*c* = 26.3574 (6) Å	µ = 0.40 mm^−^^1^
β = 96.998 (1)°	*T* = 173 K
*V* = 1514.96 (7) Å^3^	Block, colourless
*Z* = 4	0.39 × 0.37 × 0.18 mm

### Data collection

**Table:**

Bruker SMART APEXII CCD diffractometer	3785 independent reflections
Radiation source: rotating anode	3252 reflections with *I* > 2σ(*I*)
Graphite multilayer monochromator	*R*_int_ = 0.033
Detector resolution: 10.0 pixels mm^-1^	θ_max_ = 28.4°, θ_min_ = 1.6°
φ and ω scans	*h* = −11→11
Absorption correction: multi-scan (*SADABS*; Bruker, 2009)	*k* = −8→8
*T*_min_ = 0.379, *T*_max_ = 0.746	*l* = −35→35
25926 measured reflections	

### Refinement

**Table:**

Refinement on *F*^2^	Primary atom site location: structure-invariant direct methods
Least-squares matrix: full	Secondary atom site location: difference Fourier map
*R*[*F*^2^ > 2σ(*F*^2^)] = 0.035	Hydrogen site location: difference Fourier map
*wR*(*F*^2^) = 0.097	H-atom parameters constrained
*S* = 1.04	*w* = 1/[σ^2^(*F*_o_^2^) + (0.0493*P*)^2^ + 0.5625*P*] where *P* = (*F*_o_^2^ + 2*F*_c_^2^)/3
3785 reflections	(Δ/σ)_max_ = 0.001
202 parameters	Δρ_max_ = 0.32 e Å^−^^3^
0 restraints	Δρ_min_ = −0.36 e Å^−^^3^

### Special details

**Table:**

Geometry. All esds (except the esd in the dihedral angle between two l.s. planes) are estimated using the full covariance matrix. The cell esds are taken into account individually in the estimation of esds in distances, angles and torsion angles; correlations between esds in cell parameters are only used when they are defined by crystal symmetry. An approximate (isotropic) treatment of cell esds is used for estimating esds involving l.s. planes.
Refinement. Refinement of F^2^ against ALL reflections. The weighted R-factor wR and goodness of fit S are based on F^2^, conventional R-factors R are based on F, with F set to zero for negative F^2^. The threshold expression of F^2^ > 2sigma(F^2^) is used only for calculating R-factors(gt) etc. and is not relevant to the choice of reflections for refinement. R-factors based on F^2^ are statistically about twice as large as those based on F, and R- factors based on ALL data will be even larger.

### Fractional atomic coordinates and isotropic or equivalent isotropic displacement parameters (Å^2^)

**Table:**

	*x*	*y*	*z*	*U*_iso_*/*U*_eq_	
Cl1	0.46121 (5)	0.19905 (8)	0.429671 (15)	0.04483 (13)	
S1	0.26452 (4)	0.26977 (6)	0.640843 (13)	0.02803 (11)	
O1	0.10204 (12)	0.71057 (17)	0.54903 (4)	0.0332 (2)	
O2	0.28126 (13)	0.07207 (17)	0.61832 (4)	0.0362 (3)	
O3	0.16362 (12)	0.29082 (19)	0.67929 (4)	0.0375 (3)	
C1	0.20875 (16)	0.4421 (2)	0.59217 (5)	0.0287 (3)	
C2	0.25155 (16)	0.4327 (2)	0.54104 (5)	0.0293 (3)	
C3	0.33886 (17)	0.3021 (3)	0.51474 (5)	0.0319 (3)	
H3	0.3865	0.1836	0.5303	0.038*	
C4	0.35222 (18)	0.3548 (3)	0.46470 (6)	0.0341 (3)	
C5	0.28443 (18)	0.5286 (3)	0.44089 (6)	0.0372 (4)	
H5	0.2989	0.5579	0.4065	0.045*	
C6	0.19629 (18)	0.6592 (3)	0.46656 (6)	0.0349 (4)	
C7	0.18327 (17)	0.6027 (3)	0.51659 (5)	0.0312 (3)	
C8	0.11908 (17)	0.6100 (2)	0.59483 (5)	0.0309 (3)	
C9	0.1233 (2)	0.8500 (3)	0.44321 (7)	0.0446 (4)	
H9A	0.0190	0.8598	0.4516	0.067*	
H9B	0.1221	0.8448	0.4060	0.067*	
H9C	0.1813	0.9700	0.4567	0.067*	
C10	0.0382 (2)	0.7033 (3)	0.63504 (6)	0.0372 (4)	
H10A	0.0465	0.6131	0.6650	0.056*	
H10B	−0.0691	0.7220	0.6219	0.056*	
H10C	0.0837	0.8365	0.6448	0.056*	
C11	0.44638 (16)	0.3512 (2)	0.66842 (5)	0.0278 (3)	
C12	0.56259 (17)	0.2089 (3)	0.67374 (5)	0.0311 (3)	
H12	0.5468	0.0759	0.6595	0.037*	
C13	0.70350 (17)	0.2617 (3)	0.70020 (6)	0.0367 (4)	
C14	0.72140 (19)	0.4592 (3)	0.71960 (6)	0.0439 (4)	
H14	0.8165	0.4980	0.7376	0.053*	
C15	0.6050 (2)	0.6008 (3)	0.71351 (6)	0.0429 (4)	
H15	0.6211	0.7349	0.7271	0.052*	
C16	0.46468 (19)	0.5483 (3)	0.68772 (6)	0.0351 (3)	
H16	0.3837	0.6443	0.6834	0.042*	
C17	0.82931 (19)	0.1065 (4)	0.70778 (7)	0.0511 (5)	
H17A	0.9268	0.1745	0.7057	0.077*	
H17B	0.8128	0.0013	0.6812	0.077*	
H17C	0.8304	0.0424	0.7415	0.077*	

### Atomic displacement parameters (Å^2^)

**Table:**

	*U*^11^	*U*^22^	*U*^33^	*U*^12^	*U*^13^	*U*^23^
Cl1	0.0518 (3)	0.0524 (3)	0.0326 (2)	−0.0124 (2)	0.01411 (17)	−0.00595 (18)
S1	0.02839 (18)	0.0322 (2)	0.02295 (17)	−0.00829 (14)	0.00096 (13)	0.00455 (13)
O1	0.0371 (6)	0.0327 (6)	0.0284 (5)	−0.0061 (5)	−0.0012 (4)	0.0043 (4)
O2	0.0403 (6)	0.0312 (6)	0.0350 (6)	−0.0092 (5)	−0.0041 (4)	0.0018 (5)
O3	0.0322 (5)	0.0509 (7)	0.0306 (5)	−0.0060 (5)	0.0078 (4)	0.0114 (5)
C1	0.0315 (7)	0.0314 (8)	0.0224 (6)	−0.0078 (6)	0.0004 (5)	0.0034 (6)
C2	0.0317 (7)	0.0327 (8)	0.0224 (6)	−0.0120 (6)	−0.0007 (5)	0.0025 (6)
C3	0.0352 (7)	0.0346 (8)	0.0255 (7)	−0.0095 (6)	0.0010 (6)	0.0012 (6)
C4	0.0354 (8)	0.0416 (9)	0.0252 (7)	−0.0142 (7)	0.0035 (6)	−0.0027 (6)
C5	0.0414 (8)	0.0472 (10)	0.0220 (7)	−0.0186 (7)	−0.0006 (6)	0.0044 (6)
C6	0.0381 (8)	0.0377 (9)	0.0266 (7)	−0.0159 (7)	−0.0054 (6)	0.0065 (6)
C7	0.0327 (7)	0.0339 (8)	0.0256 (7)	−0.0106 (6)	−0.0022 (5)	0.0011 (6)
C8	0.0327 (7)	0.0331 (8)	0.0257 (7)	−0.0099 (6)	−0.0008 (5)	0.0034 (6)
C9	0.0520 (10)	0.0433 (10)	0.0357 (8)	−0.0131 (8)	−0.0065 (7)	0.0141 (8)
C10	0.0398 (8)	0.0368 (9)	0.0351 (8)	−0.0045 (7)	0.0045 (6)	0.0006 (7)
C11	0.0291 (7)	0.0374 (8)	0.0171 (6)	−0.0102 (6)	0.0031 (5)	0.0007 (6)
C12	0.0312 (7)	0.0407 (9)	0.0218 (6)	−0.0072 (6)	0.0057 (5)	0.0023 (6)
C13	0.0288 (7)	0.0585 (11)	0.0236 (7)	−0.0089 (7)	0.0058 (5)	0.0071 (7)
C14	0.0363 (8)	0.0699 (13)	0.0248 (7)	−0.0221 (9)	0.0005 (6)	−0.0006 (8)
C15	0.0510 (10)	0.0486 (10)	0.0291 (8)	−0.0212 (9)	0.0045 (7)	−0.0085 (7)
C16	0.0407 (8)	0.0394 (9)	0.0257 (7)	−0.0081 (7)	0.0063 (6)	−0.0026 (6)
C17	0.0306 (8)	0.0782 (14)	0.0450 (10)	−0.0011 (9)	0.0062 (7)	0.0143 (10)

### Geometric parameters (Å, º)

**Table:**

Cl1—C4	1.7432 (17)	C9—H9B	0.9800
S1—O2	1.4358 (12)	C9—H9C	0.9800
S1—O3	1.4381 (11)	C10—H10A	0.9800
S1—C1	1.7323 (15)	C10—H10B	0.9800
S1—C11	1.7684 (14)	C10—H10C	0.9800
O1—C8	1.3663 (17)	C11—C12	1.382 (2)
O1—C7	1.3770 (19)	C11—C16	1.386 (2)
C1—C8	1.361 (2)	C12—C13	1.398 (2)
C1—C2	1.4453 (19)	C12—H12	0.9500
C2—C7	1.385 (2)	C13—C14	1.389 (3)
C2—C3	1.393 (2)	C13—C17	1.503 (3)
C3—C4	1.382 (2)	C14—C15	1.380 (3)
C3—H3	0.9500	C14—H14	0.9500
C4—C5	1.396 (2)	C15—C16	1.386 (2)
C5—C6	1.387 (3)	C15—H15	0.9500
C5—H5	0.9500	C16—H16	0.9500
C6—C7	1.387 (2)	C17—H17A	0.9800
C6—C9	1.501 (2)	C17—H17B	0.9800
C8—C10	1.481 (2)	C17—H17C	0.9800
C9—H9A	0.9800		
			
O2—S1—O3	118.80 (7)	C6—C9—H9C	109.5
O2—S1—C1	108.19 (7)	H9A—C9—H9C	109.5
O3—S1—C1	108.28 (7)	H9B—C9—H9C	109.5
O2—S1—C11	107.75 (7)	C8—C10—H10A	109.5
O3—S1—C11	107.25 (7)	C8—C10—H10B	109.5
C1—S1—C11	105.85 (7)	H10A—C10—H10B	109.5
C8—O1—C7	107.04 (12)	C8—C10—H10C	109.5
C8—C1—C2	107.67 (13)	H10A—C10—H10C	109.5
C8—C1—S1	126.92 (11)	H10B—C10—H10C	109.5
C2—C1—S1	125.41 (12)	C12—C11—C16	122.28 (14)
C7—C2—C3	119.83 (13)	C12—C11—S1	118.15 (12)
C7—C2—C1	104.51 (14)	C16—C11—S1	119.34 (12)
C3—C2—C1	135.67 (14)	C11—C12—C13	119.72 (16)
C4—C3—C2	116.10 (15)	C11—C12—H12	120.1
C4—C3—H3	122.0	C13—C12—H12	120.1
C2—C3—H3	122.0	C14—C13—C12	117.86 (16)
C3—C4—C5	123.30 (16)	C14—C13—C17	121.82 (16)
C3—C4—Cl1	118.52 (14)	C12—C13—C17	120.31 (17)
C5—C4—Cl1	118.18 (11)	C15—C14—C13	121.88 (15)
C6—C5—C4	121.16 (14)	C15—C14—H14	119.1
C6—C5—H5	119.4	C13—C14—H14	119.1
C4—C5—H5	119.4	C14—C15—C16	120.38 (17)
C5—C6—C7	114.67 (15)	C14—C15—H15	119.8
C5—C6—C9	123.41 (14)	C16—C15—H15	119.8
C7—C6—C9	121.90 (16)	C11—C16—C15	117.87 (17)
O1—C7—C2	110.66 (12)	C11—C16—H16	121.1
O1—C7—C6	124.40 (15)	C15—C16—H16	121.1
C2—C7—C6	124.94 (16)	C13—C17—H17A	109.5
C1—C8—O1	110.12 (13)	C13—C17—H17B	109.5
C1—C8—C10	134.83 (14)	H17A—C17—H17B	109.5
O1—C8—C10	115.05 (14)	C13—C17—H17C	109.5
C6—C9—H9A	109.5	H17A—C17—H17C	109.5
C6—C9—H9B	109.5	H17B—C17—H17C	109.5
H9A—C9—H9B	109.5		
			
O2—S1—C1—C8	148.58 (13)	C9—C6—C7—O1	1.2 (2)
O3—S1—C1—C8	18.60 (15)	C5—C6—C7—C2	0.7 (2)
C11—S1—C1—C8	−96.14 (14)	C9—C6—C7—C2	−177.79 (15)
O2—S1—C1—C2	−32.04 (14)	C2—C1—C8—O1	−0.48 (16)
O3—S1—C1—C2	−162.03 (12)	S1—C1—C8—O1	178.99 (11)
C11—S1—C1—C2	83.23 (14)	C2—C1—C8—C10	179.12 (16)
C8—C1—C2—C7	0.71 (16)	S1—C1—C8—C10	−1.4 (3)
S1—C1—C2—C7	−178.77 (11)	C7—O1—C8—C1	0.05 (16)
C8—C1—C2—C3	−179.82 (16)	C7—O1—C8—C10	−179.64 (13)
S1—C1—C2—C3	0.7 (3)	O2—S1—C11—C12	−12.65 (13)
C7—C2—C3—C4	0.6 (2)	O3—S1—C11—C12	116.33 (12)
C1—C2—C3—C4	−178.85 (15)	C1—S1—C11—C12	−128.22 (12)
C2—C3—C4—C5	0.4 (2)	O2—S1—C11—C16	172.73 (11)
C2—C3—C4—Cl1	179.49 (11)	O3—S1—C11—C16	−58.29 (13)
C3—C4—C5—C6	−0.9 (2)	C1—S1—C11—C16	57.16 (13)
Cl1—C4—C5—C6	−179.97 (12)	C16—C11—C12—C13	1.2 (2)
C4—C5—C6—C7	0.3 (2)	S1—C11—C12—C13	−173.25 (11)
C4—C5—C6—C9	178.77 (15)	C11—C12—C13—C14	−1.0 (2)
C8—O1—C7—C2	0.43 (16)	C11—C12—C13—C17	177.98 (14)
C8—O1—C7—C6	−178.69 (14)	C12—C13—C14—C15	0.3 (2)
C3—C2—C7—O1	179.73 (13)	C17—C13—C14—C15	−178.72 (15)
C1—C2—C7—O1	−0.69 (16)	C13—C14—C15—C16	0.4 (2)
C3—C2—C7—C6	−1.2 (2)	C12—C11—C16—C15	−0.6 (2)
C1—C2—C7—C6	178.42 (14)	S1—C11—C16—C15	173.82 (11)
C5—C6—C7—O1	179.67 (13)	C14—C15—C16—C11	−0.2 (2)

### Hydrogen-bond geometry (Å, º)

Cg1 and Cg2 are the centroids of the C2–C7 benzene ring and the C1/C2/C7/O1/C8 furan ring, respectively.

**Table:**

*D*—H···*A*	*D*—H	H···*A*	*D*···*A*	*D*—H···*A*
C10—H10*C*···O2^i^	0.98	2.49	3.297 (2)	139
C17—H17*A*···O3^ii^	0.98	2.41	3.369 (2)	165

Symmetry codes: (i) *x*, *y*+1, *z*; (ii) *x*+1, *y*, *z*.
